# Invisible staffing churn in nursing homes: CMS turnover metrics miss a growing short-term workforce

**DOI:** 10.1093/haschl/qxag094

**Published:** 2026-04-18

**Authors:** Stephen Petterson, Katherine M Winter, J William Kerns, Danya M Qato, Linda Wastila, Nicole Brandt, Yu-Hua Fu, Roy T Sabo, YoonKyung Chung, Adam J Funk, Alex H Krist, Jonathan D Winter

**Affiliations:** Department of Family Medicine and Population Health, Virginia Commonwealth University School of Medicine, Richmond, VA 23224, United States; Shenandoah University School of Nursing, Winchester, VA 22601, United States; Department of Family Medicine and Population Health, Virginia Commonwealth University School of Medicine, Richmond, VA 23224, United States; Shenandoah Valley Family Practice Residency, Front Royal, VA 22630, United States; Department of Practice, Sciences, and Health Outcomes Research, University of Maryland School of Pharmacy, Baltimore, MD 21201, United States; Department of Practice, Sciences, and Health Outcomes Research, University of Maryland School of Pharmacy, Baltimore, MD 21201, United States; Department of Practice, Sciences, and Health Outcomes Research, University of Maryland School of Pharmacy, Baltimore, MD 21201, United States; Peter Lamy Center on Drug Therapy and Aging, Baltimore, MD 21201, United States; Department of Practice, Sciences, and Health Outcomes Research, University of Maryland School of Pharmacy, Baltimore, MD 21201, United States; Department of Family Medicine and Population Health, Virginia Commonwealth University School of Medicine, Richmond, VA 23224, United States; Harvey L. Neiman Health Policy Institute, Reston, VA 20191, United States; Department of Family Medicine and Population Health, Virginia Commonwealth University School of Medicine, Richmond, VA 23224, United States; Department of Family Medicine and Population Health, Virginia Commonwealth University School of Medicine, Richmond, VA 23224, United States; Department of Family Medicine and Population Health, Virginia Commonwealth University School of Medicine, Richmond, VA 23224, United States; Shenandoah Valley Family Practice Residency, Front Royal, VA 22630, United States

**Keywords:** nursing home, long-term care, staffing, turnover, stability, instability, quality-measure

## Abstract

**Introduction:**

Federal policy mandates adequate nursing home (NH) staffing, yet staffing adequacy remains difficult to define and measure. In 2022, the Centers for Medicare & Medicaid Services (CMS) incorporated annual turnover into Five-Star Ratings but adopted a definition excluding staff below a 120-hour-in-90-days threshold, potentially underestimating turnover and weakening validity.

**Methods:**

Using Payroll-Based Journal and CareCompare data (2020Q2-2024Q1), we replicated CMS-reported turnover and constructed an inclusive measure counting new hires. We assessed divergence between definitions, associations with ten standardized CMS quality indicators, and changes in facility rankings.

**Results:**

By 2022-2023, 45% of nursing hires were excluded under CMS's definition. As short-term staffing increased, CMS-specification and inclusive turnover diverged (correlation 0.91-0.82). Associations with quality outcomes were modest and similar across definitions. However, facility rankings differed substantially: only 30% of facilities remained in the same turnover decile, with reclassification concentrated among NHs with high short-term attrition, greater contract use, and distinct ownership and payer mix.

**Conclusion:**

CMS turnover metrics miss nearly half of turnover, understating instability and reshaping facility comparisons without improving associations with quality outcomes. As short-term staffing expands, the CMS measure risks becoming less informative about workforce instability, underscoring how metric definitions shape oversight and reporting.

## Teaser

Staffing instability is widely recognized as a threat to nursing home quality, and in 2022, CMS added annual staff turnover to the Nursing Home Five-Star Rating System, signaling that workforce stability is central to quality assessment. CMS defines turnover to emphasize retention of established staff, excluding workers who log fewer than 120 hours in 90 days. Yet the validity of this definitional choice has received limited scrutiny. Using Payroll-Based Journal data, we show that short-tenure workers excluded from CMS turnover reporting now account for nearly half of all new hires. By narrowing the turnover construct to longer-tenure staff, the CMS definition omits substantial workforce volatility without strengthening associations between turnover and quality outcomes. At the same time, it materially changes which facilities appear relatively stable or unstable, with reclassification concentrated among homes with high short-term attrition, greater contract staffing, and distinct ownership and payer mix. As reliance on short-term staffing grows, the CMS turnover measure may miss an increasing share of meaningful workforce churn, potentially distorting how facility stability is assessed in public reporting and oversight and demonstrating the tradeoff between emphasizing retention among longer-tenure staff and capturing meaningful workforce instability, as well as how these design decisions shape which workers are included in measurement.

Key points

- **CMS turnover reporting omits a strikingly large and growing share of the nursing home workforce**. By 2022-2023, nearly half of all new hires worked too few hours to be captured by the CMS turnover metric, leaving a substantial portion of staffing churn unreported and highlighting a major shift in workforce composition not reflected in existing public reporting.
- **Turnover definitions materially affect which facilities appear stable or unstable**. Facility placement within the turnover distribution differed substantially by turnover definition, with reclassification concentrated among homes with high short-term staffing attrition, greater reliance on contract staff, higher staffing instability, and distinct ownership and payer-mix profiles.
- **Excluding short-term staff does not improve the CMS turnover metric's relationship to quality**. CMS-replicated and inclusive turnover measures showed similarly modest adjusted associations with nursing home quality outcomes, indicating that narrowing the measure to only longer-tenure staff omits a large and growing share of workforce instability without improving performance measurement. These findings underscore that turnover metrics reflect design decisions that shape how staffing stability is assessed and compared in policy and public reporting.

## Introduction

The Nursing Home Reform Act of 1987, enforced by the Centers for Medicare & Medicaid Services (CMS), mandates that nursing homes (NHs) employ sufficient staff to provide each resident with the “necessary care and services to attain or maintain the highest practicable physical, mental, and psychosocial well-being.” Translating this mandate into operational standards and routine practice has proven challenging, however, and nearly 4 decades later, staffing sufficiency remains difficult to define, measure, and enforce. This ambiguity has contributed to substantial variation in staffing levels across NHs, with pronounced differences between facilities with the highest and lowest levels of staffing.^1-6^

Concerns about staffing adequacy have intensified in recent years, driven by rising resident acuity, persistent workforce shortages, increased reliance on contract staffing following the COVID-19 pandemic, and growing variation in access to care.^7-9^ In response, CMS has sought to improve transparency and oversight by strengthening staffing data collection.^1^ Beginning in 2017, NHs were required to submit daily staffing data each quarter through the Payroll-Based Journal (PBJ) system, using information drawn directly from payroll records.^10^ These data have substantially expanded the empirical basis for assessing staffing practices and adequacy.^11,12^ PBJ data now inform the Five-Star NH Quality Rating System and support expanded publicly reported measures of staffing adequacy.^13^

Since its introduction in 2008, the Five-Star system has included a composite staffing domain intended to capture workforce adequacy. Initially, this domain focused on staffing hours per resident day for registered nurses (RNs) and for all nursing staff. In July 2022, CMS expanded the staffing composite to include PBJ-derived measures of weekend staffing and annual turnover for long-term RNs, total nursing staff, and administrators, explicitly recognizing that workforce continuity, in addition to staffing levels, is central to care quality.^1,13-16^ The inclusion of turnover measures represented an important conceptual advance, as high attrition among core staff can disrupt care continuity, weaken staff–resident relationships, and erode safety culture, even when nominal staffing levels appear adequate.^17,18^

However, CMS's current definition for calculating RN and total nursing turnover has important implications for how workforce stability is measured and compared across facilities. The CMS turnover measure is intentionally restricted to long-term staff, defined as those who accumulate at least 120 hours during their first 90 days of employment, and excludes staff who do not meet this threshold. The given rationale is to remove “individuals who work infrequently (eg, occasionally covering shifts at a nursing home).’^1^ The 120-hour criterion, however, also excludes contract staff working intermittently as well as regular staff who quit shortly after their start date. As a result, facilities with a highly churning short-term workforce may paradoxically appear stable, reporting low turnover under the CMS definition despite frequent changes in the staff providing hands-on care to residents. This measurement design choice may obscure staffing instability and complicate interpretation of turnover-based comparisons across facilities.

The broader consequences of defining turnover around long-tenure employees while omitting short-term staff remain uncertain. Although associations between staffing levels and NH quality are well established, it is less clear how this turnover definition affects the measure's validity and usefulness for assessing staffing stability, evaluating adequacy, and predicting care quality.^3,7,8,19-23^ Focusing only on longer-tenure staff may understate turnover in facilities that rely heavily on short-hour employment and may systematically alter how facilities are classified relative to one another. Whether this design choice enhances the measure's ability to assess association with care quality remains an open question.

This study addresses these gaps by using PBJ data to replicate the CMS turnover algorithm for total nursing staff and RNs and comparing it with an otherwise identical, more inclusive measure that counts all new hires regardless of hours worked. We quantify the share of turnover omitted under the CMS definition, examine trends in short-term employment, and assess how often facilities change their relative turnover rankings when calculated under the 2 approaches. Because this reclassification can affect the publicly reported staffing measures that inform consumer choice, oversight, and quality-improvement efforts, understanding the magnitude and repercussions of these shifts is important. We therefore identify facility and staffing characteristics associated with such patterns and compare the predictive performance of alternative turnover measures for resident quality outcomes. More broadly, this study examines how varying definitions of turnover shape measured staffing instability, facility comparisons, and associations with quality outcomes. In the context of public reporting and oversight, definitional choices directly influence how staffing metrics are interpreted and applied in NHs. Our analysis clarifies and highlights the implications of these design decisions.

## Data and methods

### Data and study sample

We constructed a national, facility-level dataset using multiple administrative and publicly available sources. Daily staffing data were obtained from CMS's PBJ Employee Detail files, which report hours worked by individual employees by job category and contract status. PBJ data include daily staffing hours for certified nursing assistants (CNAs), licensed practical nurses (LPNs), and RNs. PBJ data include daily staffing hours for certified nursing assistants (CNAs), LPNs, and RNs. Employee-level PBJ files providing daily, worker-specific staffing records are publicly available beginning in 2020Q2 and are complete and nationally consistent from that point forward. These files are more detailed than the aggregated PBJ Nurse Staffing files, reported by staff category, that have been available since 2017Q1.^10,24^ Facilities were excluded based on predefined data completeness and observation criteria, including missing or incomplete PBJ reporting, inability to link across data sources, or insufficient consecutive quarters to construct turnover measures using the CMS six-quarter framework. Across the study period, missing data rates ranged from a peak of 22% (3326/15 393) in 2020q4 to a low of 13% (1967/15 030) in 2023Q1.

Facility characteristics, quality measure outcomes, and CMS-reported turnover rates were obtained from Nursing Home Care Compare files.^13^ Resident case mix and payer mix were drawn from LTCFocus using the most recent available year (2023) and CMS Medicare cost reports.^25,26^ Neighborhood characteristics were merged at the ZIP Code Tabulation Area (ZCTA) level using American Community Survey (ACS) 5-year estimates (2019-2023).^27^ Because LTCFocus and ACS variables do not fully correspond with PBJ data, the most recent available values were carried forward under the assumption that neighborhood characteristics do not change substantially over short time horizons.

### Measures

CMS publicly reports annual turnover rates for nursing staff calculated using a standardized methodology. Separate rates are reported for total nursing staff and for RNs. Each turnover rate is calculated using 6 consecutive calendar quarters: a baseline quarter (Q0), 4 observation quarters (Q1–Q4), and a trailing quarter (Q5) (Appendix Figure S1). Under this framework, to calculate the turnover rate associated with Q1, the denominator identifies all periods of employment that existed in Q0 or began in Q1–Q2, and the numerator counts the number of those spells that ended during Q1–Q4, with Q5 used to confirm temporary separations occurring near the end of the observation period (see Appendix Figure S1).^1^ Using PBJ daily staffing data, we replicated the CMS turnover methodology for each facility and each six-quarter window beginning in 2020Q2.^1,14^

In the CMS specification, staff are included in the denominator only if they accumulated at least 120 hours during their first 90 days after the inferred start date.^13^ We replicated this algorithm using PBJ data to generate what we refer to as CMS-specification turnover. We also constructed a more inclusive turnover measure that retained the CMS framework but counted all inferred new hires regardless of hours worked during the first 90 days. Under this inclusive definition, the six-quarter window structure and separation rules were unchanged. Throughout the analyses, we distinguish among *CMS-reported* turnover from Care Compare, *CMS-specification* turnover derived from PBJ data, and the PBJ-based *inclusive* turnover measure that removes the 120-hours-in-90-days inclusion rule.

### Statistical analysis

We conducted 4 complementary analyses: (1) correlations between turnover definitions; (2) regression models of quality outcomes with bootstrap coefficient comparisons; (3) decile reclassification; and (4) multinomial models of reclassification correlates.

First, we examined correlations between our replication of the CMS turnover measure, the officially reported CMS turnover rates, and turnover rates calculated using the inclusive definition.

Second, we estimated facility-level mixed-effects models relating turnover (CMS-specification vs inclusive) to ten CMS quality outcomes, including long-stay measures of increased need for help with activities of daily living, antipsychotic use, worsened independent mobility, and pressure ulcers; short-stay measures of new antipsychotic use, mobility decline, rehospitalization, and emergency department visits; and utilization measures of hospitalizations and emergency department visits per 1000 long-stay resident-days, adjusting for facility, market, and staffing covariates and including quarter indicators and a facility random intercept. Covariates included facility characteristics (size, ownership, chain affiliation, and occupancy), payer mix, market concentration, neighborhood sociodemographic characteristics, contract staffing use, and measures of staffing instability and adequacy. All measures were obtained from Nursing Home CareCompare and aligned with the corresponding turnover observation periods. To directly compare the 2 turnover definitions, we used a facility-level block bootstrap (1000 resamples of 1000 facilities, with all quarters retained) and, within each resample, re-estimated paired models and computed the difference in turnover coefficients; we report the median difference and 95% percentile interval.

Third, to assess how turnover definitions affect relative facility position within the turnover distribution, NHs were classified into deciles based on their turnover rates under each definition. Using data associated with the 2023Q1-2023Q4 turnover measures, facilities were assigned to deciles separately under the CMS-specification and inclusive measures. These classifications were cross-tabulated to generate a reclassification matrix, in which diagonal cells represent unchanged decile placement, and off-diagonal cells represent changes when short-term staff are included.

Finally, to identify facility characteristics associated with reclassification across total nursing turnover measures, we estimated a multinomial logistic regression model with 3 mutually exclusive outcome categories: no reclassification, upward reclassification, and downward reclassification. Facilities with no reclassification were assigned to the same turnover decile under both definitions. Upward reclassification indicates placement in a lower turnover decile under the CMS-specification measure than under the inclusive measure, whereas downward reclassification indicates placement in a higher turnover decile. All models included controls for facility characteristics. We also incorporated staffing stability and adequacy measures. High contract use was defined as being in the upper half of the facility distribution of the percentage of total hours worked by contract staff, providing a simple and interpretable distinction between facilities with relatively higher vs lower reliance on contract staffing, consistent with prior approaches to categorizing staffing patterns.^11,28^ Staffing instability was defined as the percentage of days in the prior year on which staffing hours fell below 80% of the facility's mean daily hours per resident day and was modeled using quartiles, with the most stable quartile as the reference group; this threshold follows prior work on staffing instability in NHs and captures meaningful deviations from typical staffing levels.^11,28^

Mixed-effects models (and bootstrap resamples) were estimated in SAS 9.4 using PROC GLIMMIX; all other analyses were conducted in Stata 19.5.

### Analytic scope and exclusions

We excluded hospital-based facilities and NHs that failed CMS Payroll-Based Journal (PBJ) data quality checks, including missing quarterly submissions or inconsistent employee identifiers, consistent with CMS guidance.

Analyses varied by objective. Reclassification and multinomial models were restricted to turnover rates constructed using the CMS six-quarter rolling window beginning in 2023Q2 (Appendix Figure S1) to reflect the most recent observation period. Correlation and trend analyses used all available rolling six-quarter turnover windows beginning in 2020Q3 through 2023Q2.

## Results

**Figure 1:**
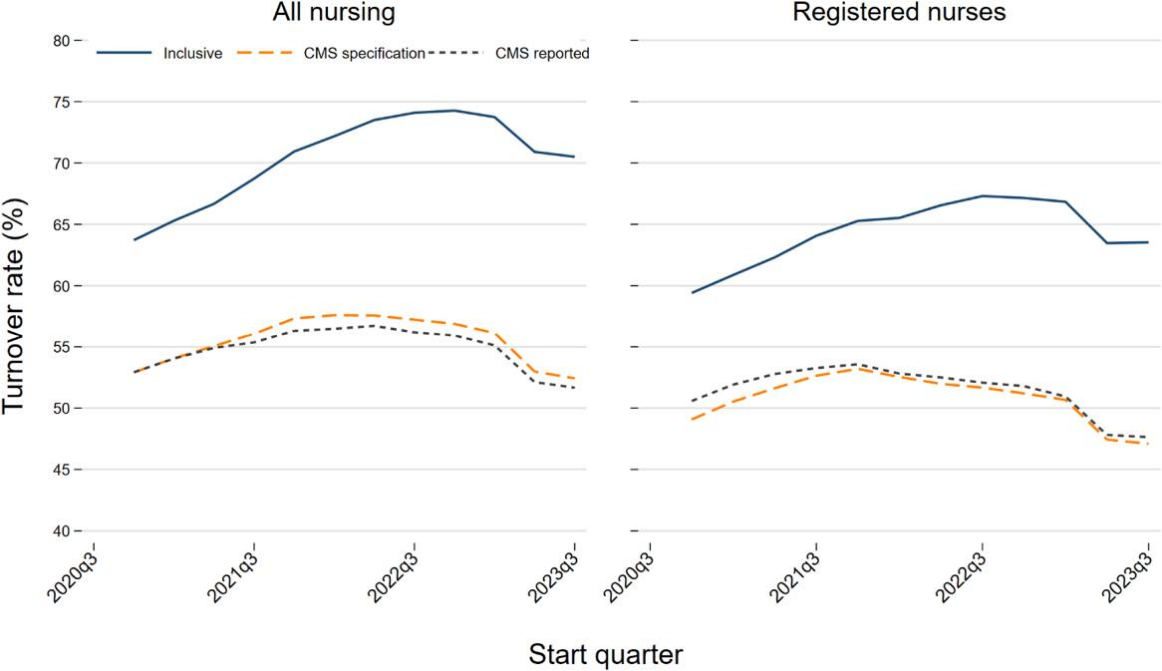
Mean annual nursing home turnover rates under CMS-reported, CMS-specification, and inclusive definitions, 2020Q3-2023Q3. Notes: Each point represents the unweighted mean of facility turnover rates calculated for the six-quarter window beginning in the labeled start quarter (see Appendix Figure S1 for window structure). “CMS-reported” turnover is obtained from Nursing Home Care Compare. “CMS-specification” turnover reflects our PBJ-based implementation of the CMS algorithm, including the 120-hours-in-90-days inclusion rule. “Inclusive” turnover applies the same algorithm but counts all inferred new hires regardless of hours worked during the first 90 days.

PBJ-derived turnover calculated using the CMS specification closely matched CMS-reported turnover throughout the study period (Figure 1). Correlations between our replication of CMS's RN turnover measure and CMS-reported RN turnover exceeded 0.95 and approached 1.0 in the earliest years. Total nursing turnover showed similarly high and stable correlations over time, confirming high fidelity of the PBJ-based implementation of the CMS-specification algorithm. Descriptive statistics for turnover and quality measures across the study period are shown in Appendix Table S1.

In contrast, correlations between the CMS-specification and inclusive turnover measures declined steadily over time (Figure 1). Early in the study period, facility-level correlations between the 2 measures were approximately 0.90 for both RNs and total nursing staff. By 2023, correlations had fallen to approximately 0.82. This divergence coincided with growth in short-tenure employment that is excluded by design from the CMS-specification measure (Appendix Figure S2). By the end of the study period, 46% of total nursing hires and 41% of RN hires worked fewer than 120 hours in their first 90 days and were therefore excluded under the CMS specification. Quarterly mean turnover under the CMS specification closely tracked CMS-reported turnover (Appendix Figure S3), whereas inclusive turnover was consistently higher and the gap widened over time. By late 2022-2023, inclusive turnover exceeded CMS-specification turnover by roughly 19% points for all nursing staff and 16% points for RNs.

Associations with quality outcomes were nearly identical across definitions, despite differences in measured turnover levels (Table 1). A 1 SD increase in turnover was associated with worse outcomes under both measures. For example, inclusive turnover was associated with higher odds of increased need for help with activities of daily living (OR 1.082; 95% CI, 1.079-1.085) and worsened independent mobility (OR 1.085; 95% CI, 1.081-1.089); corresponding CMS-specification estimates were 1.081 (95% CI, 1.078-1.084) and 1.083 (95% CI, 1.079-1.087). Utilization outcomes were similar, including outpatient emergency department visits per 1000 long-stay resident-days (inclusive RR 1.079; 95% CI, 1.075-1.083; CMS-specification RR 1.085; 95% CI, 1.082-1.089). Differences in associations between turnover definitions and quality outcomes were small and attenuated with covariate adjustment. Bootstrap comparisons confirmed that coefficient differences were centered near zero; in adjusted models, differences ranged from −0.008 to 0.003 on the log scale, with confidence intervals spanning zero (Appendix Table S2).

**Table 1:** Adjusted associations between quality outcomes and CMS-specification and inclusive turnover measures.

	Exponentiated coefficients, exp(β) (95% CI)		Bootstrap results
Quality measure	Inclusive turnover measure	CMS turnover measure	Median difference in coefficients (β_INCL − β_CMS) (95% CI)
**Measured as proportions [0-1]**			
Need for help with daily activities has increased (long stay)	1.082 (1.079, 1.085)	1.081 (1.078, 1.084)	0.001 (−0.012, 0.013)
Receiving an antipsychotic medication (long stay)	0.998 (0.994, 1.002)	1.006 (1.002, 1.010)	−0.008 (−0.025, 0.011)
Residents who newly received an antipsychotic medication (short stay)	1.035 (1.028, 1.042)	1.038 (1.031, 1.044)	−0.003 (−0.027, 0.022)
Ability to move independently worsened (long stay)	1.085 (1.081, 1.089)	1.083 (1.079, 1.087)	0.002 (−0.013, 0.017)
Residents with pressure ulcers (long stay)	1.073 (1.070, 1.077)	1.071 (1.067, 1.075)	0.003 (−0.013, 0.017)
Declined or stayed the same in their ability to move around on their own (short stay)	1.016 (1.012, 1.021)	1.019 (1.014, 1.024)	−0.002 (−0.023, 0.018)
Rehospitalized after a nursing home admission (short stay)	1.025 (1.023, 1.027)	1.025 (1.023, 1.027)	0.000 (−0.008, 0.008)
Outpatient emergency department visit (short stay)	1.057 (1.054, 1.061)	1.060 (1.056, 1.063)	−0.002 (−0.014, 0.010)
**Measured as rates**			
Number of hospitalizations per 1000 long-stay resident days (long stay)	1.020 (1.018, 1.023)	1.021 (1.018, 1.023)	−0.001 (−0.011, 0.010)
Number of outpatient emergency department visits per 1000 long-stay resident-days (long stay)	1.079 (1.075, 1.083)	1.085 (1.082, 1.089)	−0.006 (−0.020, 0.009)

The first 2 columns report exponentiated coefficients [exp(β)] from adjusted regression models relating facility turnover (inclusive or CMS-specification) to ten quality outcomes. Proportion outcomes were estimated using fractional logit models; rate outcomes were estimated using log-linked Poisson models. Models included facility random effects, quarter indicators, and covariate adjustment as described in Methods. Estimates correspond to a one–standard deviation increase in the turnover measure. The final column reports the median difference in coefficients (β_INCL − β_CMS) and 95% confidence intervals from 1000 facility-level bootstrap resamples. Differences are reported on the log scale.

**Figure 2:**
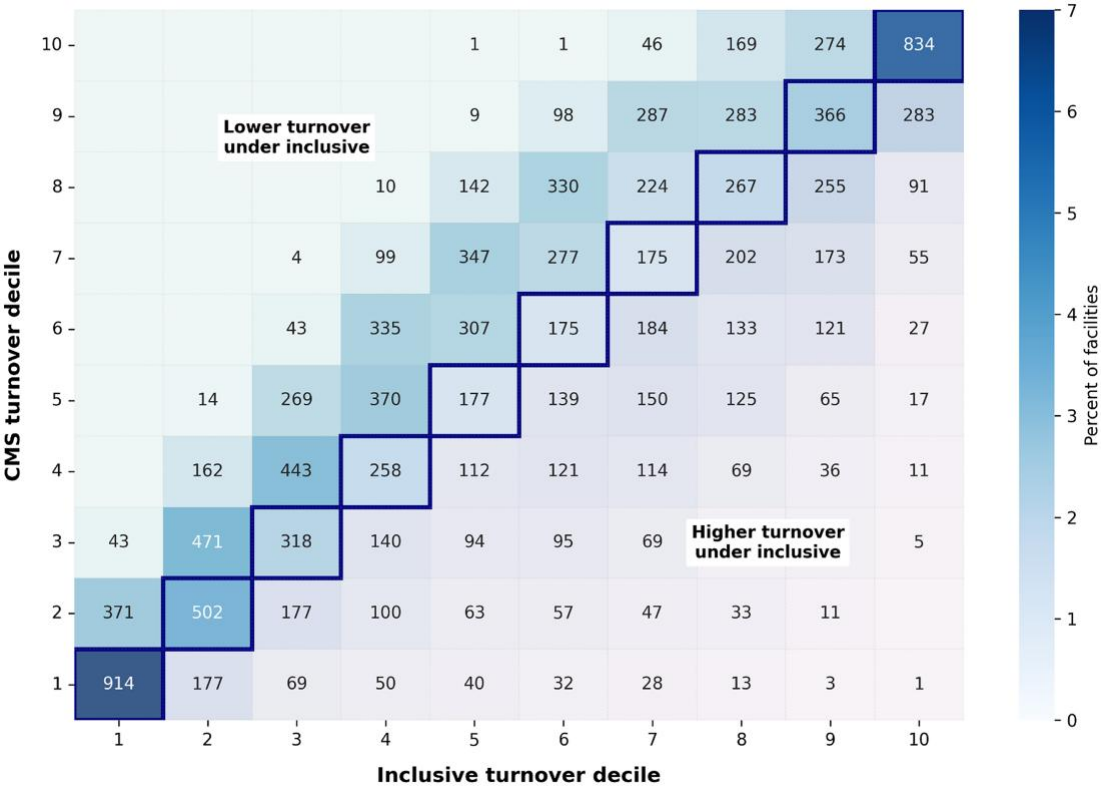
Reclassification of nursing homes across turnover deciles under CMS-specification and inclusive measures, 2023Q1. Notes: Each cell shows the number of nursing homes classified into the corresponding CMS-specification turnover decile (rows) and inclusive turnover decile (columns) for the six-quarter window beginning 2023Q1. The outlined diagonal indicates facilities assigned to the same decile under both definitions. Shading reflects the percentage of facilities in each cell. Deciles are constructed separately for each turnover definition using facility-level turnover rates.

Although outcome associations were similar, facility classification differed substantially by turnover definition (Figure 2). When facilities were grouped into turnover deciles using rates constructed from the six-quarter window beginning in 2023Q2, only 30% remained in the same decile under both definitions. Most facilities shifted deciles when inclusive rather than CMS-specification turnover was used, and many moved by 2 or more deciles. Facilities with greater short-term staffing churn tended to appear in lower-turnover deciles under the CMS-specification measure but in higher-turnover deciles under the inclusive definition.

Multinomial regression analyses indicated that reclassification was systematic rather than random (Table 2). Facilities with greater reliance on contract staffing were substantially more likely to be reclassified to higher turnover deciles under the inclusive definition; facilities with more than 20% contract staffing had a relative risk ratio (RRR) of 3.45 for upward reclassification compared with facilities reporting no contract staff. In contrast, facilities in the highest staffing instability quartile (>6.7% of days below 80% of mean staffing) were more likely to be reclassified downward (RRR 1.33). Structural and market characteristics were also associated with reclassification patterns. For-profit and chain-affiliated facilities were more likely to shift upward, whereas larger, higher-occupancy facilities and those in more concentrated markets were more likely to shift downward.

**Table 2:** Facility characteristics associated with reclassification to higher or lower turnover under the inclusive turnover definition.

Facility characteristic	Higher turnover vs no reclassification (RRR)	Lower turnover vs no reclassification (RRR)
For-profit ownership	1.171*** (0.056)	0.984 (0.046)
Log(number of beds)	0.638*** (0.031)	1.265*** (0.065)
Chain status (ref: not in chain)		
Small chain	1.395*** (0.074)	1.089* (0.055)
Medium chain	1.384*** (0.080)	1.047 (0.057)
Large chain	1.545*** (0.090)	0.954 (0.051)
Occupancy rate	0.468*** (0.062)	1.916*** (0.241)
Rural	0.862** (0.050)	1.080 (0.054)
HHI	0.528*** (0.064)	1.402*** (0.126)
Log median household income	1.213*** (0.076)	0.867** (0.053)
Percent Medicaid resident days	0.671*** (0.058)	1.079 (0.086)
Percent foreign-born population	0.613*** (0.089)	1.475*** (0.196)
Contract staffing share (ref: no contract staff)		
0.1%-20%	3.161*** (0.159)	0.608*** (0.025)
20%	3.453*** (0.201)	0.453*** (0.024)
Staffing instability quartile (ref: 0%)		
0.1%-2.2%	1.006 (0.052)	1.171*** (0.059)
2.3%-6.7%	0.909* (0.049)	1.311*** (0.066)
6.7%	0.654*** (0.039)	1.329*** (0.070)
Observations	24 132	24 132

RRR denotes relative risk ratios from a multinomial logistic regression model. The reference outcome is no reclassification. Higher (lower) reclassification indicates assignment to a higher (lower) turnover decile under the inclusive definition relative to the CMS-specification measure. RRRs greater than 1 indicate a higher relative likelihood of reclassification vs no reclassification for the specified facility characteristic. Robust standard errors are shown in parentheses. HHI denotes the Herfindahl–Hirschman Index. Models include the full set of facility and market covariates described in Methods. *P < 0.05, **P < 0.01, ***P < 0.001.

## Discussion

As NH staffing patterns have shifted toward shorter periods of employment and greater reliance on flexible and contract labor, the CMS turnover measure has become less aligned with the workforce dynamics it is intended to capture.^13^ In 2022-2023, nearly half of newly hired nursing staff fell below the 120-hour threshold and were therefore excluded from the CMS-specification turnover measure. This design choice removes a substantial share of workforce instability from public reporting and has widened the gap between the CMS-specification and inclusive turnover definitions.

CMS's use of a minimum-hours threshold reflects a purposeful effort to focus turnover measurement on retention of core staff and to exclude workers who contribute only intermittently to facility staffing.^1^ This approach may have been more appropriate in earlier labor market contexts, when short-tenure and contract staffing were less prevalent. However, current staffing patterns include a large and growing share of workers with short employment durations. As a result, hours-based inclusion rules now exclude a substantial portion of the workforce that contributes to day-to-day care delivery.

In sensitivity analyses applying alternative thresholds of 60, 90, and 150 hours in the first 90 days, reclassification remained substantial across all specifications: even under the most permissive threshold (60 hours), only 40% of facilities remained in the same turnover decile, and more than one in 5 shifted by 2 or more deciles (Appendix Table S3). This pattern indicates that any minimum-hours rule would exclude a substantial segment of short-tenure staff, suggesting that the observed divergence reflects a structural limitation of threshold-based definitions rather than a specific parameter choice. Thus, the choice of threshold becomes less about fine-tuning measurement and more about determining which segments of the workforce are visible in public reporting.

Short-term staff contribute meaningfully to workforce instability. Early attrition is particularly consequential in direct-care roles, where continuity is central to quality and frequent cycling of staff can disrupt resident routines, strain remaining workers, and complicate care coordination. By restricting turnover measurement to longer-tenure staff, the CMS specification excludes a large and growing segment of employment that increasingly characterizes the contemporary NH workforce.

At the same time, restricting turnover measurement to longer-tenure staff does not strengthen its relationship with resident outcomes. Across a broad range of quality and utilization measures, CMS-specification and inclusive turnover exhibited nearly identical adjusted associations. Differences observed in unadjusted models attenuated with covariate adjustment, and bootstrap comparisons confirmed that coefficient differences were small and centered near zero. Restricting turnover measurement to longer-tenure staff therefore removes information about a substantial and expanding segment of the workforce without improving the measure's ability to capture meaningful variation in quality. Collectively, these findings suggest that differences between turnover definitions primarily influence how facilities are classified and compared, rather than the strength of their associations with measured outcomes, a distinction with important implications for how staffing stability is reflected in public reporting.

As a result, the principal consequence of the 120-hour rule is comparative distortion. Facility placement within the turnover distribution differed markedly by definition, with only about 30% of NHs occupying the same turnover decile under both measures. Reclassification was systematic rather than random. These shifts reflected consistent differences in staffing practices and broader facility and market characteristics, indicating that the CMS definition systematically reshapes how workforce stability is characterized across NHs.

At a conceptual level, these findings highlight a policy tension embedded in turnover measurement: whether turnover metrics should prioritize retention of longer-tenure staff or capture instability across the full workforce. CMS's current definition emphasizes continuity by focusing on longer-tenure employees. However, short-tenure and contract staff now represent a substantial and growing share of the workforce and contribute meaningfully to day-to-day care delivery. Excluding these workers therefore narrows the construct of turnover in ways that may no longer align with contemporary staffing models.

To better reflect current workforce dynamics, CMS could consider alternative strategies for measuring turnover. One option would be to report parallel measures, one capturing retention among longer-tenure staff and another capturing total workforce churn. Another option would be to stratify turnover by employment type, distinguishing between directly employed and contract staff. A third option would be to adopt an hours-weighted turnover measure, in which separations are weighted by the number of hours worked, allowing staff with greater involvement in care delivery to have proportionally greater influence on the measure while still capturing turnover among short-tenure or part-time workers. These approaches could preserve the conceptual value of measuring continuity while avoiding the systematic exclusion of short-tenure staff. Fundamentally, these choices underscore a key insight for quality assessment: turnover metrics are not neutral descriptors of staffing stability but constructed measures that reflect policy decisions about which workers are included in assessments of facility performance.

Taken together, these findings suggest that the CMS turnover measure risks losing relevance as an indicator of workforce instability in a staffing environment increasingly defined by short-term employment. As turnover metrics assume a larger role in public reporting and regulatory oversight, definitions that overlook short-term staffing dynamics risk obscuring critical information needed to assess staffing conditions and compare facility performance.^29^

### Limitations

Our study has several limitations. Analyses rely on CMS administrative data, including PBJ and Care Compare files, which may contain reporting inconsistencies or variation in classification of contract and pooled workers. Periods of employment were inferred from observed days worked using the CMS algorithm and may misclassify intermittent or irregular work patterns despite adherence to the published specification.

Reclassification analyses were anchored to a single recent turnover window to isolate the effect of the 120-hour inclusion rule; although this clarifies definitional differences, it does not capture seasonal or outbreak-related variation in staffing dynamics. This study period includes the COVID-19 pandemic, which was associated with substantial disruptions to staffing patterns, regulatory oversight, and reporting processes in NHs. These changes may have increased variability in both staffing and quality measures and could attenuate observed associations between turnover and resident outcomes. However, because our analysis compares alternative turnover definitions within the same time period, both measures are subject to the same underlying conditions, supporting the internal validity of our comparisons. Although we could not directly reconstruct Five-Star ratings, the substantial decile reclassification we document suggests that turnover definition may meaningfully alter facilities’ placement within the staffing domain of the Five-Star system, with potential implications for consumer choice and regulatory oversight. Quantifying these effects is an important area for future evaluation. Associations between turnover and quality outcomes are observational and may reflect unmeasured confounding, including leadership practices, local labor market conditions, or resident case mix not fully captured in available covariates.

While we adjusted for facility-level measures of resident case mix using LTCFocus data, we did not directly adjust outcome measures for resident-level acuity or risk, which may contribute to residual confounding. Analyses were conducted at the facility level and cannot assess staff- or resident-level mechanisms. Finally, although our PBJ-based replication closely tracked CMS-reported turnover rates, minor discrepancies remain, likely reflecting operational details or data-processing procedures not publicly documented.

Despite these constraints, to our knowledge, this study is the first to fully operationalize the CMS turnover specification using publicly available PBJ data. By replicating the federal algorithm and comparing it with an otherwise identical inclusive definition, we provide a transparent and reproducible framework for evaluating how turnover metric design influences assessments of workforce stability and facility performance. The divergence observed between definitions reflects deliberate design choices rather than implementation error, establishing a foundation for future policy evaluation and benchmarking.

## Conclusion

Short-term staffing now represents a large and growing share of the NH workforce. Yet the CMS turnover metric excludes staff who work fewer than 120 hours in their first 90 days, increasingly omitting meaningful workforce instability from public reporting and systematically understating volatility in facility staffing. This exclusion does not improve associations with resident outcomes, but it substantially reshapes how facilities are classified, often making homes with high levels of short-term or contract staffing appear more stable than they are in practice.

As turnover metrics play an expanding role in Five-Star ratings, consumer decision-making, and regulatory oversight, measurement choices matter. Definitions that narrow turnover to longer-tenure staff risk becoming progressively less informative in a labor market characterized by short term and flexible staffing employment. Refining turnover measurement to better reflect contemporary workforce dynamics is feasible using existing PBJ data and would improve transparency, enhance comparability across facilities, and better align public reporting with the staffing conditions experienced by residents.

## Supplementary Material

qxag094_Supplementary_Data
