# Supplementary material for: Invisible staffing churn in nursing homes: CMS turnover metrics miss a growing short-term workforce
Source: Health Aff Sch. 2026 Apr 18;4(5):qxag094. doi: 10.1093/haschl/qxag094 (PMC13143168; doi:10.1093/haschl/qxag094)
Supplement: qxag094_Supplementary_Data [file qxag094_supplementary_data.zip › Staffing paper 1 appendixes 4-14.docx]

**Appendix Figure A1. Six-Quarter Window Used to Calculate CMS Turnover (Denominator and Numerator Periods)**


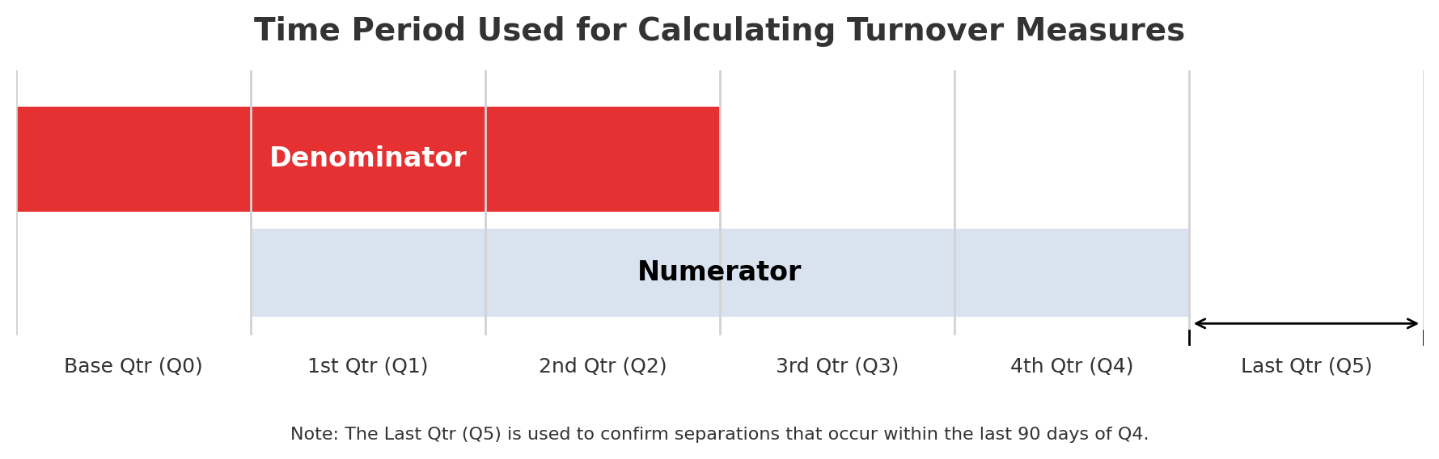


**Notes:** This figure reproduces the six-quarter rolling structure described in the CMS Design for Care Compare Nursing Home Five-Star Quality Rating System: Technical Users Guide (2025, p. 14). Under the CMS specification, turnover is calculated using a baseline quarter (Q0), four observation quarters (Q1–Q4), and a trailing quarter (Q5). The denominator includes periods of employment that existed in Q0 or began in Q1–Q2. The numerator includes separations from those spells occurring during Q1–Q4. Q5 is used to confirm separations occurring near the end of Q4. Periods of employment that both began and ended in Q0 are excluded from the denominator, consistent with CMS guidance.

**Appendix Figure A2. Share of Newly Hired Nursing Staff Excluded from the CMS Turnover Measure Under the 120-Hour-in-90-Days Rule, 2020Q3–2023Q3**
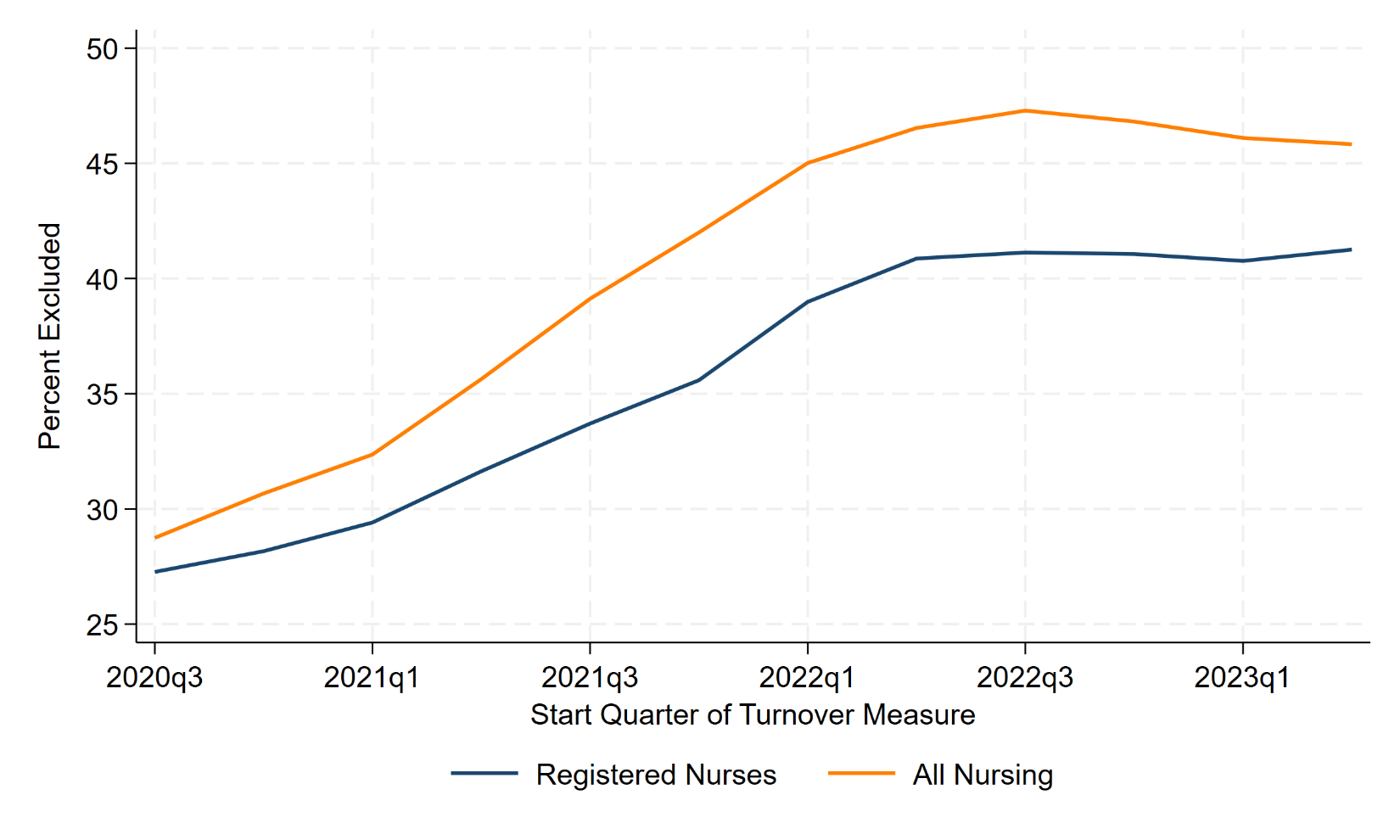


**Notes**: The figure displays the percentage of newly hired staff who worked fewer than 120 hours during their first 90 days of employment and were excluded from the CMS turnover measure under the 120-hour-in-90-days inclusion rule. Each point corresponds to the starting quarter of the six-quarter turnover window. Data are from CMS Payroll-Based Journal files.

**Appendix Figure A3. Facility-Level Correlations Among CMS-Reported, CMS-Specification, and Inclusive Turnover Measures, 2020Q3–2023Q3**
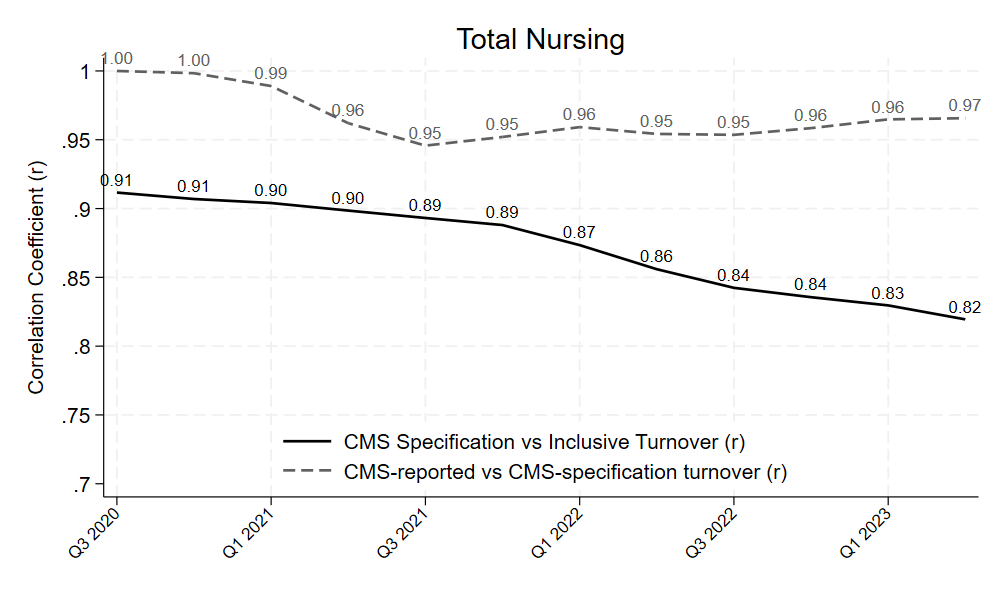


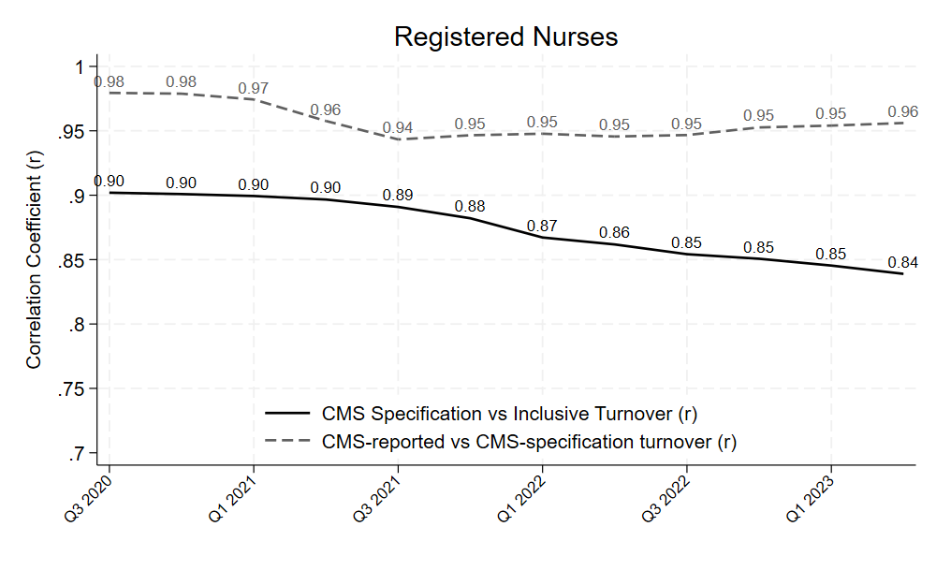


**Notes**: Each point represents the Pearson correlation across facilities for turnover rates calculated over the six-quarter window beginning in the labeled start quarter. The dashed line shows correlations between CMS-reported turnover from Care Compare and our PBJ-based replication of the CMS specification. The solid line shows correlations between the CMS-specification and inclusive turnover measures.

**Appendix Table A1. Descriptive Statistics for Turnover and Quality Measures, 2020Q2–2024Q1**

|  | **Mean (Quarterly Min, Max)** | **SD (Quarterly Min, Max)** |
| --- | --- | --- |
| **Turnover Measures** |  |  |
| CMS-specification | 0.525 (0.512, 0.534) | 0.152 (0.148, 0.158) |
| Inclusive | 0.653 (0.611, 0.675) | 0.167 (0.157, 0.171) |
| **Quality Measures** |  |  |
| Need for help with daily activities has increased (Long Stay) | 0.150 (0.139, 0.168) | 0.068 (0.066, 0.070) |
| Receiving an Antipsychotic Medication (Long Stay) | 0.143 (0.141, 0.145) | 0.090 (0.088, 0.092) |
| Short Stay Residents Who Newly Received an Antipsychotic Medication | 0.015 (0.014, 0.016) | 0.015 (0.014, 0.016) |
| Ability to Move Independently Worsened (Long Stay) | 0.177 (0.147, 0.248) | 0.093 (0.076, 0.106) |
| Residents with pressure ulcers (Long Stay) | 0.081 (0.078, 0.084) | 0.045 (0.044, 0.046) |
| Declined or stayed the same in their ability to move around on their own (Short Stay) | 0.247 (0.214, 0.287) | 0.117 (0.113, 0.119) |
| Rehospitalized after a nursing home admission (Short Stay) | 0.223 (0.217, 0.229) | 0.068 (0.063, 0.071) |
| Outpatient emergency department visit (Short Stay) | 0.109 (0.094, 0.125) | 0.057 (0.049, 0.063) |
| Number of hospitalizations per 1000 long-stay resident-days (Long Stay) | 1.601 (1.397, 1.827) | 0.747 (0.649, 0.821) |
| Number of outpatient emergency department visits per 1,000 long-stay resident-days (Long Stay) | 0.907 (0.702, 1.169) | 0.625 (0.481, 0.759) |

**Notes**: Statistics are calculated at the facility-quarter level. The overall mean and standard deviation reflect pooled values across all facilities and quarters in the analytic sample. Minimum and maximum quarter values refer to the lowest and highest quarterly means (or standard deviations) observed across the study period. Proportion measures are bounded between 0 and 1; utilization measures are rates per 1,000 resident-days.

| **Appendix Table A2. Unadjusted Associations Between Quality Outcomes and CMS-Specification and Inclusive Turnover Measures** | | | | | | |  |  |  |
| --- | --- | --- | --- | --- | --- | --- | --- | --- | --- |
|  | **Exponentiated Coefficients [exp(β)] (95% CI)** | | | |  | **Bootstrap Results** | | |  |
| **Quality Measure** | | **Inclusive turnover measure** | **CMS turnover measure** |  | **Median Difference in Coefficients (β_INCL − β_CMS) (95% CI)** | | |  |  |
| **Measured as Proportions [0-1]** | |  |  |  |  |  | | | |
| Need for help with daily activities has increased (Long Stay) | | 1.103 (1.099, 1.106) | 1.107 (1.103, 1.110) |  | -0.004 (-0.017, 0.009) | | |  |  |
| Receiving an Antipsychotic Medication | | 0.999 (0.995, 1.003) | 1.014 (1.010, 1.018) |  | -0.009 (-0.032, 0.015) | | |  |  |
| Short Stay Residents Who Newly Received an Antipsychotic Medication | | 1.043 (1.037, 1.050) | 1.053 (1.046, 1.059) |  | -0.015 (-0.032, 0.002) | | |  |  |
| Ability to Move Independently Worsened | | 1.072 (1.069, 1.076) | 1.075 (1.072, 1.079) |  | -0.003 (-0.018, 0.012) | | |  |  |
| Residents with pressure ulcers | | 1.062 (1.059, 1.066) | 1.066 (1.063, 1.070) |  | -0.003 (-0.019, 0.010) | | |  |  |
| Declined or stayed the same in their ability to move around on their own (Short) | | 1.041 (1.036, 1.045) | 1.036 (1.031, 1.040) |  | 0.004 (-0.018, 0.028) | | |  |  |
| Rehospitalized after a nursing home admission (Short Stay) | | 1.043 (1.041, 1.045) | 1.030 (1.028, 1.033) |  | 0.012 (0.004, 0.021)* | | |  |  |
| Outpatient emergency department visit (Short Stay) | | 1.065 (1.062, 1.069) | 1.086 (1.082, 1.089) |  | -0.018 (-0.031, -0.006)* | | |  |  |
| **Measured as Rates** | |  |  |  |  | | |  |  |
| Number of hospitalizations per 1000 long-stay resident days (Long Stay) | | 1.037 (1.035, 1.040) | 1.032 (1.029, 1.035) |  | 0.005 (-0.006, 0.016) | | |  |  |
| Number of outpatient emergency department visits per 1000 long-stay resident day | | 1.090 (1.086, 1.094) | 1.120 (1.116, 1.124) |  | -0.028 (-0.043, -0.013)* | | |  |  |

**Notes**: The first two columns report odds ratios for quality measures bounded between 0 and 1 and rate ratios for utilization measures, estimated using unadjusted models. All estimates correspond to a one–standard deviation increase in the turnover measure. The final column reports the median difference in coefficients (β_INCL − β_CMS) and 95% confidence intervals from 1,000 bootstrap resamples of 1,000 facilities, retaining all quarters for sampled facilities. Values near zero indicate similar association strength across turnover definitions.

| **Appendix Table A3. Sensitivity of Turnover Reclassification to Alternative Hours-in-First-90-Days Thresholds, Total Nursing Staff** | | | | |  |
| --- | --- | --- | --- | --- | --- |
| **Minimum Hours** **Threshold** | **New Hires** **Excluded (%)** | **Correlation with** **Inclusive Turnover** | **Facilities in Same** **Turnover Decile (%)** | **Facilities Shifting** **≥2 Deciles (%)** |  |
| 60 hours | 28.3 | 0.900 | 39.7 | 21.3 |  |
| 90 hours | 33.1 | 0.867 | 35.4 | 28.4 |  |
| **120 hours (CMS)** | **36.8** | **0.843** | **32.7** | **32.9** |  |
| 150 hours | 39.9 | 0.823 | 31.2 | 36.0 |  |
|  |  |  |  |  |  |
| Notes: Each row applies an alternative minimum-hours-in-first-90-days threshold to define eligibility for the turnover denominator, holding all other elements of the CMS six-quarter algorithm constant. "New Hires Excluded" is the mean facility-level share of newly hired nursing staff falling below the threshold. "Correlation" is the Pearson correlation between threshold-specific and inclusive (all-hires) turnover across facilities. Decile reclassification compares each facility's threshold-specific turnover decile with its inclusive turnover decile. The 120-hour row (shaded) corresponds to the current CMS specification. Analysis restricted to the six-quarter window beginning 2023Q1; roster-reset facilities excluded. N = 14,167 facilities (total nursing staff). | | | | |  |
|  |  |  |  |  |  |
|  |  |  |  |  |  |
|  |  |  |  |  |  |
|  |  |  |  |  |  |
